# Self-regulated mechanism of Plk1 localization to kinetochores: lessons from the Plk1-PBIP1 interaction

**DOI:** 10.1186/1747-1028-3-4

**Published:** 2008-01-23

**Authors:** Kyung S Lee, Doo-Yi Oh, Young H Kang, Jung-Eun Park

**Affiliations:** 1 Laboratory of Metabolism, Center for Cancer Research, National Cancer Institute, National Institutes of Health, Bethesda, MD 20892, USA

## Abstract

Mammalian polo-like kinase 1 (Plk1) has been studied extensively as a critical element in regulating various mitotic events during M-phase progression. Plk1 function is spatially regulated through the targeting activity of the conserved polo-box domain (PBD) present in the C-terminal non-catalytic region. Recent progress in our understanding of Plk1 localization to the centromeres shows that Plk1 self-regulates its initial recruitment by phosphorylating a centromeric component PBIP1 and generating its own PBD-binding site. Paradoxically, Plk1 also induces PBIP1 delocalization and degradation from the mitotic kinetochores late in the cell cycle, consequently permitting itself to bind to other kinetochore components. Thus, PBIP1-dependent self-recruitment of Plk1 to the interphase centromeres serves as a prelude to the efficient delivery of Plk1 itself to other kinetochore components whose interactions with Plk1 are vital for proper mitotic progression.

## Background

**Figure 1:**
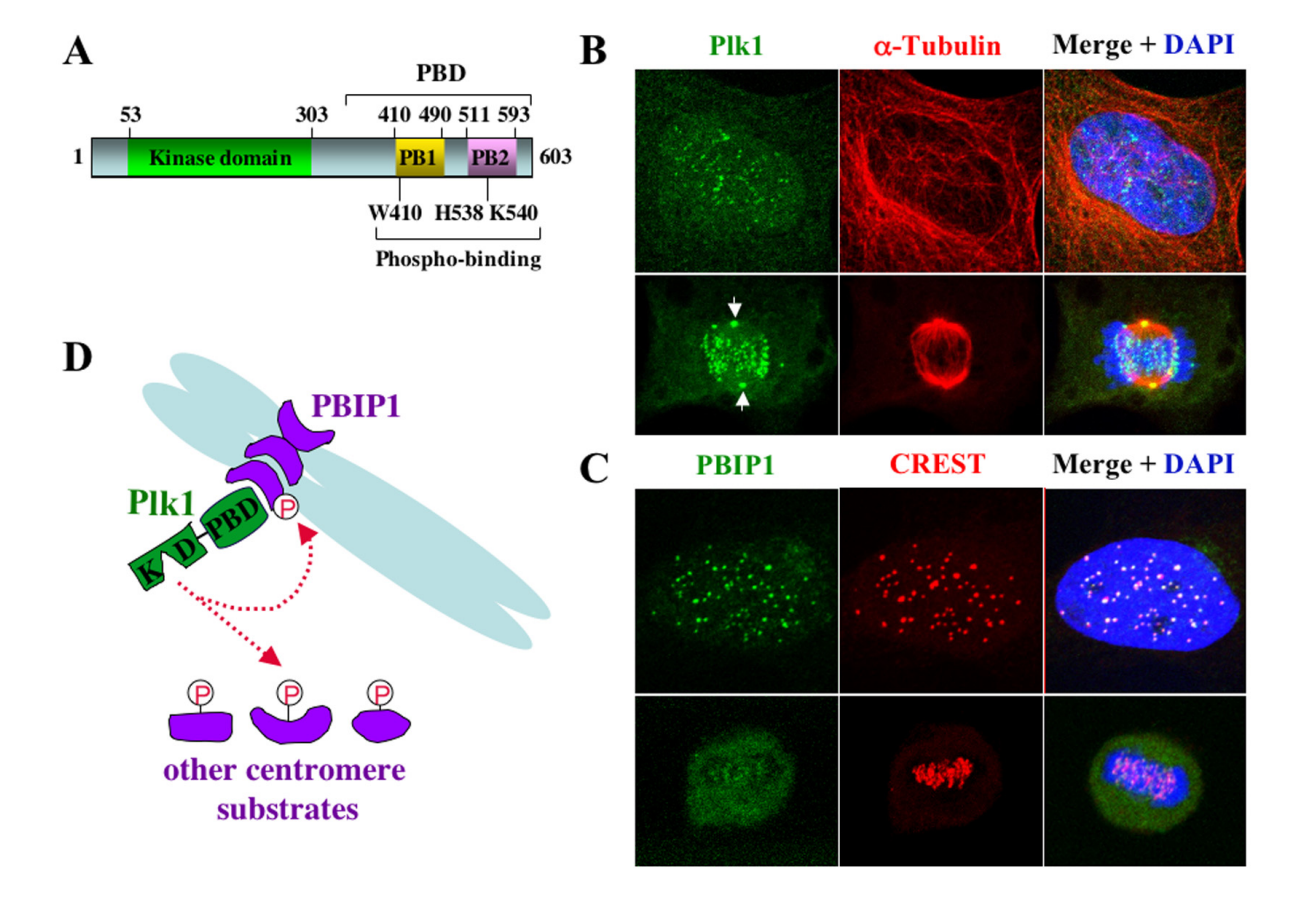
Plk1 colocalizes with PBIP1 at the interphase and mitotic centromeres. **(A)**, Structure of mammalian Plk1. Numbers indicate amino acid positions. Critical amino acid residues are shown. **(B-C)**, HeLa cells were costained with anti-Plk1 and anti-α-tubulin antibodies **(B) **or anti-PBIP1 and mouse anti-CREST antibodies **(C)**. Images were collected with a Zeiss LSM510 confocal microscope. Arrows, centrosome-localized Plk1 signals. **(D)**, Model illustrating the Plk1-PBIP1 interaction at the centromeres. PBIP1 functions as a centromeric scaffold to recruit Plk1. Once localized at the centromeres, Plk1 phosphorylates either PBIP1 or other centromeric Plk1 substrates. KD, kinase domain; PBD, polo-box domain.

Polo-like kinases (collectively, Plks) have been isolated from various species from budding yeast to mammalian cells. In addition to the N-terminal kinase domain, they are characterized by the presence of a highly conserved polo-box domain (PBD) in the C-terminal non-catalytic region that is critical for subcellular localization (Fig. 1A) (see reviews, [1,2]). In mammalian cells, multiple Plks with distinct functions appear to exist. These include Plk1, Plk2/Snk, Plk3/Prk/Fnk, and Plk4/Sak. Plk4/Sak is the most distantly-related member of the Plks subfamily and one of the two isoforms, Sak-a, possesses a significant C-terminal extension with only the PB1 motif. Among these four members, Plk1 drew most of the attention because of its tight association with oncogenesis. Studies in various organisms have shown that Plk1 and its functional homologs in lower eukaryotic organisms (*Xenopus *Plx1, *Drosophila *polo, fission yeast Plo1, and budding yeast Cdc5) play critical roles in various mitotic events such as centrosome maturation, bipolar spindle formation, APC (anaphase promoting complex) activation, and cytokinesis. The mitotic functions of Plk1 during M-phase progression and the importance of PBD for Plk1 function have been comprehensively reviewed in recent years (see reviews, [3-6]). This communication will focus on the dynamic temporal and spatial regulation of Plk1 localization to the interphase and mitotic centromeres as cells proceed through the cell cycle. Failure in this regulation results in a chromosome congression defect that ultimately leads to chromosome missegregation and aneuploidy.

### PBD, a phospho-binding module critical for subcellular localization of Plk1

Studies in cultured mammalian cells revealed that Plk1 localizes to the centrosomes as early as late S and to the centromeres in early G2. These localizations become most prominent during early mitosis and persist until late anaphase ([7-9], and Fig. 1B). In anaphase, likely due to changes in Plk1-binding proteins, Plk1 delocalizes from the centrosomes and kinetochores and relocalizes to the spindle midzone (later it becomes midbody). However, the mechanisms underlying Plk1 relocalization to specific subcellular locations and the elements critical for these events are largely elusive. A growing body of evidence suggests that PBD plays a pivotal role in targeting the catalytic activity of Plk1 to specific subcellular structures. The initial finding that demonstrated the importance of the PBD for Plk1 localization came from the analysis of PBD mutants in a genetically-amenable heterologous organism, budding yeast. A single point mutation of the conserved Trp414 in the PB1 of PBD (Fig. 1A) was sufficient to disrupt both the localization and the ability of Plk1 to functionally complement a mitotic defect associated with a mutation in the budding yeast Plk1 homolog *CDC5 *[10]. Subsequent analyses with analogous mutations in the PBD show that PBD is critical for the localization and mitotic functions of various Plks in their native organisms [9,11,12]. Consistent with these observations, a single W414F mutation is sufficient to disrupt the ability of PBD to bind to its binding target peptide [13].

Recently, PBD has been shown to bind to a phosphorylated motif [14]. Determination of the co-crystal structure of the Plk1 PBD bound to its optimal phosphopeptide provided molecular insights as to how the PBD interacts with its binding target. Results show that PBD is composed of PB1 (aa 411–489 in Plk1) and PB2 (aa 511–592 in Plk1) motifs that have identical folds of β6α (a six-stranded anti-parallel β-sheet and an α-helix). PB1 and PB2 form a hetero-dimeric phosphorecognition module of a zipper-like structure that can accommodate a phosphorylated peptide at a cleft between the two motifs [15,16]. The Trp414 and Leu490 residues from PB1 appear to be crucial for non-polar interactions with the neighboring residues of the phospho-peptide, whereas the His538 and Lys540 residues from PB2 are vital for electrostatic interactions with the negative charges of the phospho-Ser/Thr residue of the phospho-peptide. Subsequent studies show that the PBD of Plk1 binds to phosphorylated optimal peptide sequences of Ser-pThr/pSer-Pro/X (pThr/pSer, pThr or pSer; X, any amino acid) with the critical requirement for Ser at the pThr-1 position and a loose selectivity for Pro at the pThr+1 position [16]. These findings suggest that PBD likely binds to a phosphorylated target that is primarily primed by cyclin-dependent kinases (Cdks) or other Pro-directed kinases [14,16]. Since priming phosphorylation of PBD-docking proteins by another kinase promotes Plk1 interaction with these proteins or targets Plk1 in proximity with other substrates, the priming step provides an additional layer of regulation to ensure ordered Plk1 functions during M-phase progression.

### Inverse correlation between Plk1 and its PBD-binding protein, PBIP1

PBIP1/MLF1IP/KLIP1/CENP-50/CENP-U (PBIP1 hereafter) was isolated as a PBD-interacting protein from a yeast two-hybrid screening [17] and has been shown to be critical for proper recruitment of Plk1 to the interphase and mitotic centromeres [17]. PBIP1 also interacts with latent nuclear antigen (LNA) of Kaposi's sarcoma-associated herpes virus (KSHV) [18] and with MDS/myeloid leukemia factor 1 (MLF1) [19], although the physiological significance of these interactions are largely elusive. In cultured cells, PBIP1 colocalizes with kinetochore-specific CREST antigens (Fig. 1C). In addition, PBIP1 has been shown to be a centromere component important for proper chromosome segregation [17,20-22]. Since PBIP1 directly interacts and colocalizes with Plk1 at the centromeres, one simple possibility is that the PBIP1-Plk1 interaction at the centromeres targets Plk1 to PBIP1 itself and/or other centromere substrates whose phosphorylation by Plk1 is critical for interphase and mitotic events (Fig. 1D).

**Figure 2:**
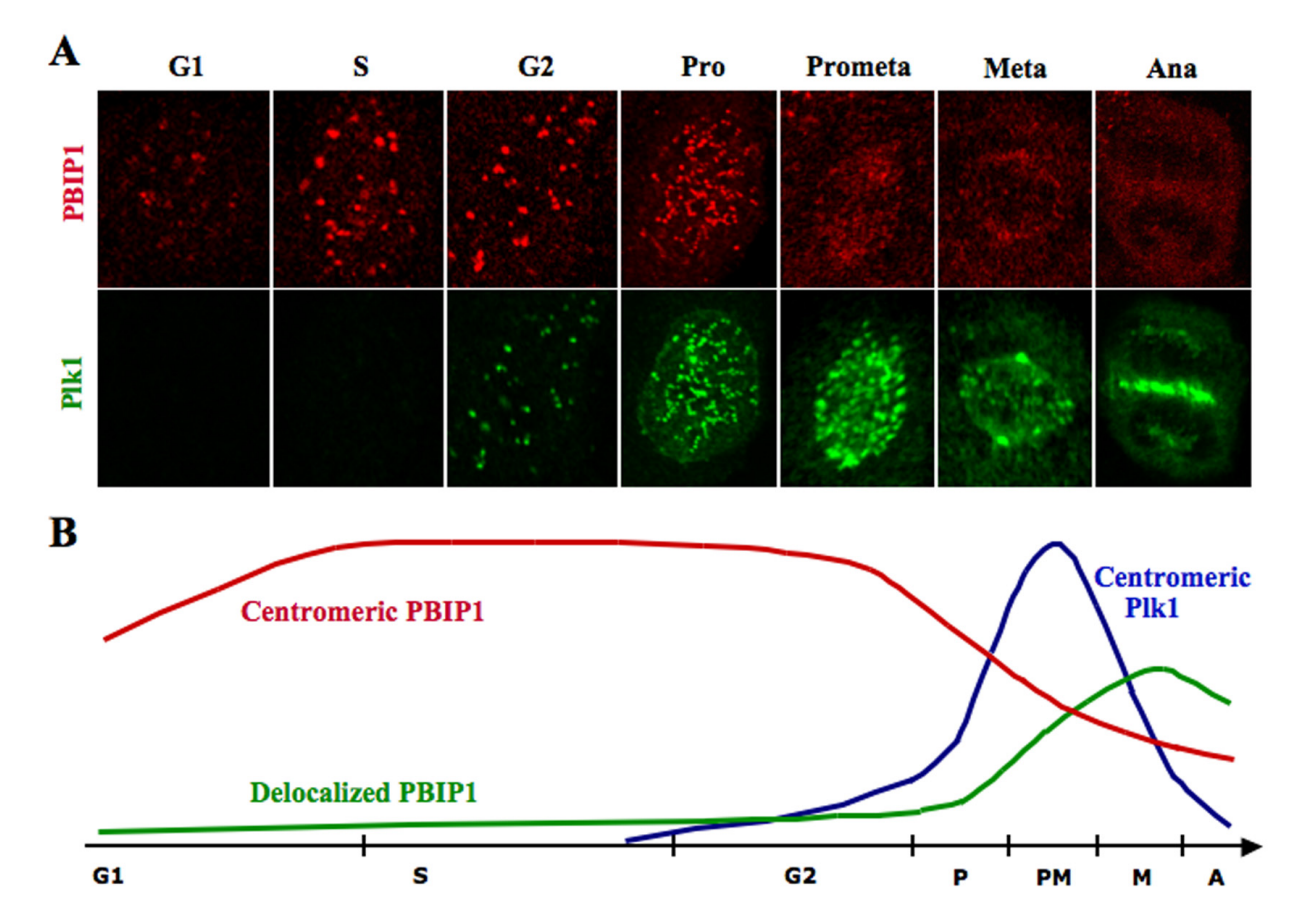
Localization patterns of PBIP1 and Plk1 during the cell cycle. **(A)**, Asynchronously growing HeLa cells were costained with anti-Plk1 and anti-PBIP1 antibodies. Representative images from each stage of the cell cycle are shown. Note that, except G2, inverse correlation between PBIP1 and Plk1 localization is manifest. **(B)**, Schematic diagram illustrating the fluctuation of PBIP1 and Plk1 fluorescence intensities at the centromeres/kinetochores during the cell cycle. During interphase, PBIP1 is tightly localized to centromeres (Red). A rise in the Plk1 level and activity (Blue) during early mitosis induces PBIP1 delocalization from the kinetochores, thus yielding an increased level of non-centromeric PBIP1 population (Green). The free Plk1 population from the p-T78 PBIP1 tether may interact with various PBD-binding proteins or substrates at or near the kinetochores.

Close examination of the PBIP1 and Plk1 localization during the cell cycle revealed that PBIP1 localizes to the interphase centromeres as early as the G1/S boundary where cells have no detectable Plk1 signals (see G1 and S phase cells in Fig. 2A), indicating that PBIP1 localization to the centromeres precedes that of Plk1. The level of centromeric PBIP1 signal is high in late interphase (S and G2), but diminishes precipitously as cells proceed through mitosis (see mitotic cells in Fig. 2A). On the other hand, Plk1 begins to localize to the centromeres in G2 and becomes most abundant at the prometaphase kinetochores before it relocalizes to midzone in anaphase (Fig. 2A). Thus, the level of PBIP1 localized at the centromeres inversely correlates with that of Plk1. Consistent with this observation, measurement of the fluorescence intensities for PBIP1 signals revealed that PBIP1 was highly centromeric (i.e., without any significant level of non-centromeric PBIP1 signals) early in the cell cycle, where Plk1 was not detectably expressed (G1 and S phases in Fig. 2B). As the level of Plk1 at the centromeres increases, the level of centromeric PBIP1 precipitously diminished later in the cell cycle (prophase and prometaphase in Fig. 2B). Reflecting the diminished level of the centromeric PBIP1 signals, the level of non-centromeric PBIP1 sharply increased as cells entered mitosis (Fig. 2B). Since Plk1 phosphorylates PBIP1, these observations hint that Plk1-dependent PBIP1 modification (i.e., phosphorylation) ultimately leads to PBIP1 delocalization from mitotic kinetochores. Consistent with this notion, depletion of Plk1 prolongs PBIP1 localization to the centromeres (Y. H. Kang and K. S. Lee, unpublished), suggesting that Plk1 negatively regulates PBIP1 localization to the centromeres.

### PBIP1 delocalization vs. degradation

**Figure 3:**
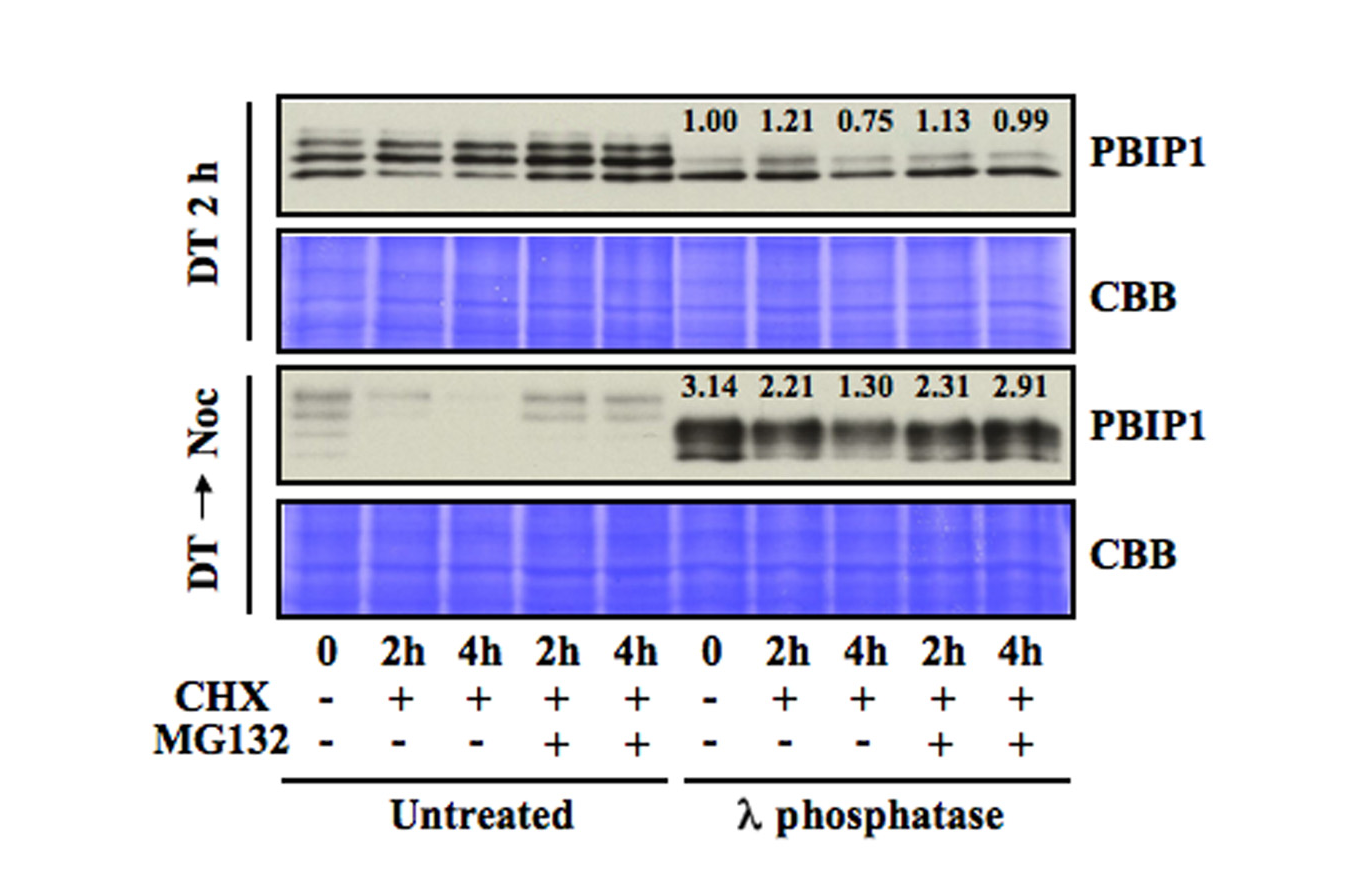
Stability of PBIP1 in S and M phases of the cell cycle. To examine the level of PBIP1 stability and to assess the degree of PBIP1 delocalization during the cell cycle, HeLa cells were arrested at the G1/S boundary by double thymidine block, and then treated as described below. **Top panel**, Cells were released from the G1/S block into fresh medium. Two hours after release (DT 2 h), cells were treated with cycloheximide (20 μM) to inhibit protein synthesis. Where indicated, cells were additionally treated with proteasome inhibitor MG132 (10 μM) to prevent PBIP1 degradation. **Bottom panel**, Cells were released from the G1/S block into nocodazole (200 ng/ml)-containing medium (DT → Noc) to trap the cells in prometaphase. Ten hours after release, cells were treated as described above. **Top and Bottom panels**, the same amount of total cellular lysates (50 μg) were treated with either 200 U of λ phosphatase for 1 h at 30°C or left untreated. Samples were mixed with SDS sample buffer, boiled, and separated by 10% SDS-PAGE. Anti-PBIP1 immunoblotting analysis was carried out at the same time for both the top and bottom samples to directly compare all the chemiluminascent signals. The same membrane was stained with Coomassie (CBB) for loading controls. Numbers at the Top and Bottom panels indicate the relative levels of PBIP1 comparison to that of the DT 2 h sample.

**Figure 4:**
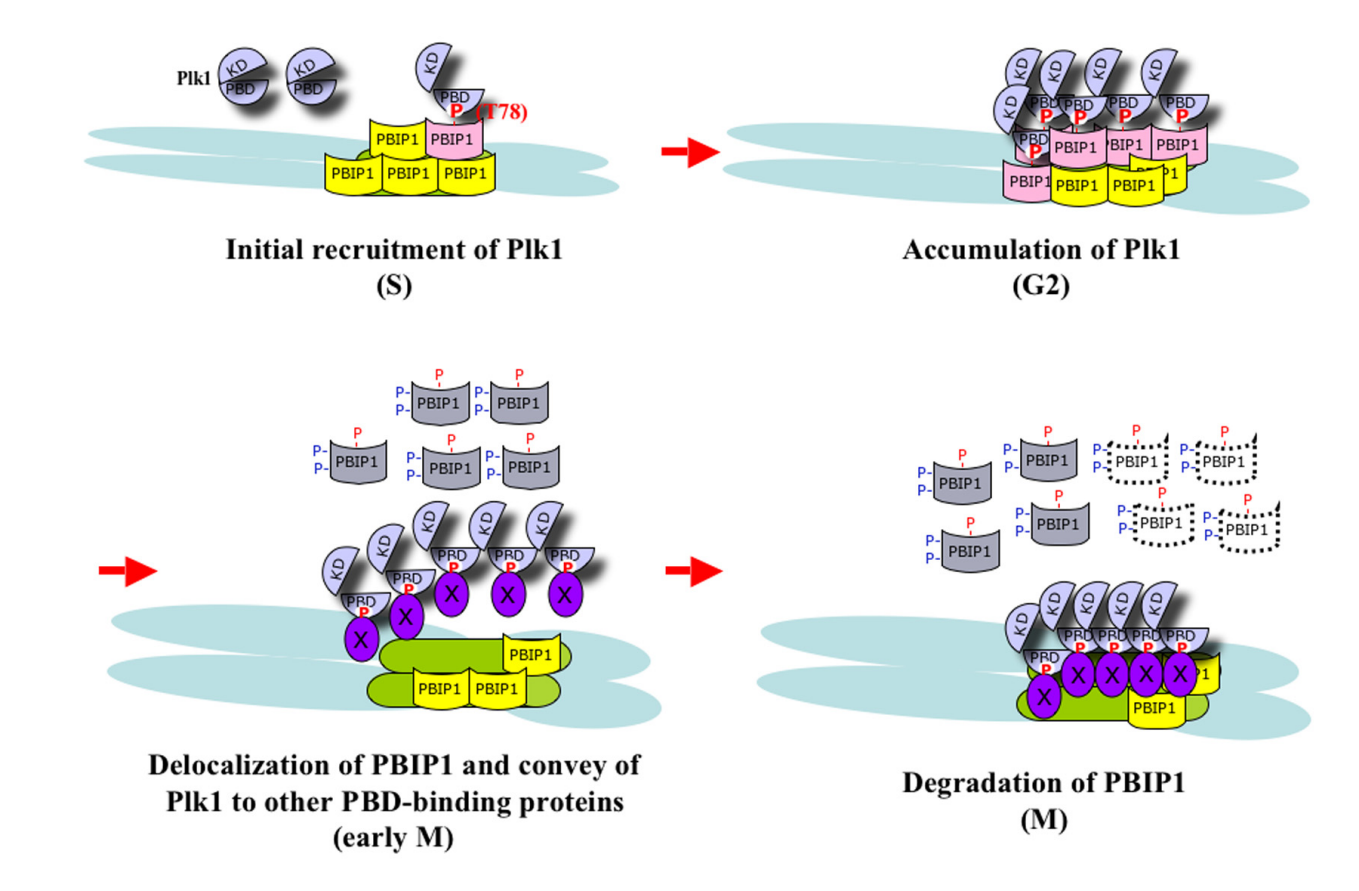
A model illustrating the self-regulated mechanism of Plk1 localization to the interphase and mitotic centromeres. Early in the cell cycle, PBIP1 accumulates at the interphase kinetochores prior to Plk1 localization (S phase). As Plk1 is expressed in G2, Plk1 interacts with PBIP1 and phosphorylates T78 to create a self-docking site for its PBD (G2 phase). This step is critical to promote its own recruitment to the kinetochores. In early mitosis, a surge in the Plk1 activity induces hyperphosphorylation and delocalization of PBIP1 from the mitotic kinetochores in a manner that is not understood at present (early M phase). As the level of PBIP1 at the kinetochore diminishes, Plk1 liberated from the p-T78 PBIP1 tether binds to other kinetochore components or substrates (marked "X") critical for proper M-phase progression (M phase).

PBIP1 is tightly localized to the interphase centromeres, but significantly delocalized from the mitotic kinetochores, generating a detectable level of non-centromeric PBIP1. In agreement with these observations, PBIP1 appeared to be largely stable in S phase (compare the 0 h sample with the 2 h or 4 h sample in Fig 3, upper panel). In prometaphase cells, however, the level of PBIP1 appeared to be greatly diminished after 4 h under nocodazole-trapped conditions (Fig. 3, lower panel, untreated). Surprisingly, treatment of the same amount of lysates with λ phosphatase led to a dramatic reappearance of a several-fold higher level of underphosphorylated PBIP1 forms (Fig. 3, lower panel, λ phosphatase-treated). These observations suggest that a majority of delocalized PBIP1 is severely modified (mostly hyperphosphorylated judging from the λ phosphatase experiment) in such a way that it is undetectable by conventional SDS-PAGE. Examination of the levels of dephosphorylated PBIP1 in the presence or absence of proteasome inhibitor MG132 revealed that ~60% of PBIP1 was degraded during the 4 h nocodazole-arrest. Thus, PBIP1 becomes abundant at the late stages (G2 and M) of the cell cycle and hyperphosphorylation of PBIP1 may induce its delocalization from the centromeres and subsequent degradation during mitosis (see Fig. 4). Recovery of a large fraction of PBIP1 by λ phosphatase treatment also suggests that proteasome-dependent degradation is a rate-limiting step. Furthermore, PBIP1 downregulation at the mitotic kinetochores occurs in a distinct two-step process – hyperphosphorylation-dependent delocalization and subsequent proteasome-dependent degradation.

### Paradoxical nature of the Plk1-PBIP1 interaction

The inverse nature of PBIP1 and Plk1 localization to the centromeres suggests that these two proteins function either antagonistically or independently for different cellular processes. However, depletion of PBIP1 disrupts Plk1 localization to the interphase centromeres and greatly impairs Plk1 localization to the mitotic kinetochores [17], suggesting that PBIP1 is critical for proper Plk1 localization to these locations during interphase and early mitosis. Further investigation on the mechanism underlying Plk1 recruitment to the centromeres revealed that, contrary to the widespread model that Plk1 binds to the phospho-epitope generated by Pro-directed kinases, Plk1 phosphorylates PBIP1 at T78 and binds to the resulting S77-p-T78 motif through the PBD [17]. These findings demonstrate that Plk1 phosphorylates and generates a self-docking site on PBIP1 to induce a stable interaction between Plk1 and PBIP1. Thus, recruitment of Plk1 to the kinetochores requires both the localized PBIP1 scaffold and the S77-p-T78 motif-dependent PBIP1-PBD interaction. This view predicts that Plk1 should bind to PBIP1 with a low affinity prior to PBIP1 phosphorylation at T78. Intriguingly, only Plk1, but not the two closely related Plk2 or Plk3, generates and binds to the S77-p-T78 motif (J. E. Park and K. S. Lee, unpublished), demonstrating the specificity of the p-T78-dependent PBIP1-Plk1 interaction.

Ironically, Plk1 also induces PBIP1 degradation in a S77-p-T78 motif-dependent manner [17], suggesting that both S77 and T78 residues that are central for PBD binding are also required for proper PBIP1 degradation. The current hypothesis is that Plk1-dependent PBIP1 phosphorylation at multiple sites after binding to the S77-p-T78 motif induces disassembly of the Plk1-PBIP1 complex and dissociation of PBIP1 from the mitotic kinetochores that ultimately leads to PBIP1 degradation during mitosis. Thus, Plk1-dependent PBIP1 phosphorylation not only promotes its own recruitment to the interphase centromeres but also triggers its own liberation from the PBIP1 scaffold at the mitotic kinetochores (Fig. 4). Then, how can Plk1 self-regulate these seemingly contradictory events at distinct stages of the cell cycle? One possibility is that each event requires different levels of Plk1 activities. A low level of Plk1 activity during interphase could be sufficient to generate the p-T78 tether for binding, but not sufficient to induce the disassembly of the Plk1-PBIP1 complex and degradation of PBIP1. Following its activation at the onset of mitosis, Plk1 may hyperphosphorylate PBIP1 to disassemble the Plk1-PBIP1 complex and delocalize PBIP1 for degradation. Alternatively, a positive factor accumulated in mitosis may bind to the Plk1-PBIP1 complex to induce PBIP1 delocalization and degradation. Since the level of the p-T78 epitope reaches a maximum level prior to PBIP1 delocalization/degradation, Plk1-dependent PBIP1 phosphorylation may serve as a signature for recruiting component(s) important for this event. In either case, these arguments suggest that a well-controlled balance between the Plk1 activity and the PBIP1 level is critical for proper Plk1 recruitment and timely elimination of PBIP1 from the mitotic kinetochores.

### PBIP1, a temporary Plk1 scaffold at the interphase and mitotic centromeres

Although it is clear that PBIP1 is critical for Plk1 recruitment to the interphase and early mitotic centromeres, Plk1 still localizes to the mitotic kinetochores even after delocalization/degradation of PBIP1 from these structures (Fig. 2). This finding suggests that the mechanism of Plk1 localization at the kinetochores likely involves a dynamic exchange of various Plk1-binding proteins. Consistent with this view, Plk1 is shown to interact with INCENP, Bub1, and BubR1 [23-26], whose localization to the kinetochores are most prominent at the prometaphase. These observations suggest that the Plk1 population freed from the Plk1-PBIP1 complex following PBIP1 delocalization/degradation interacts with INCENP, Bub1, and BubR1 and stably localizes to the mitotic kinetochores. However, it should be noted that INCENP, Bub1, and BubR1 do not localize to the interphase kinetochores, suggesting that they are not responsible for the early recruitment of Plk1 to the interphase centromeres. Thus, PBIP1 functions as a temporary scaffold crucial for the early recruitment of the centromeric Plk1 population until its delocalization/degradation allows Plk1 to interact with other kinetochore components during early mitosis. The interaction between Plk1 and INCENP or BubR1 has shown to be required for normal mitotic progression [24,25], supporting the notion that timely release of Plk1 from the PBIP1 scaffold is important for proper interaction with other kinetochore components, and therefore normal mitotic progression. In this regard, it will be interesting to examine whether a prolonged Plk1-PBIP1 interaction by a Plk1-binding competent, yet stable, PBIP1 mutant restrains the kinetochore Plk1 from interacting with other components or its substrates and interferes with its function during M-phase progression.

### Summary and perspective

In addition to the much-studied cyclin-dependent protein kinases, it is now widely appreciated that Plk1 functions as an integral component for various mitotic events. Plk1 is tightly regulated both temporally and spatially throughout the cell cycle. Timely recruitment of Plk1 to specific subcellular structures is likely important for proper function of Plk1 at these locations. A centromeric protein, PBIP1, binds to Plk1 with a high affinity and plays an important role in recruiting Plk1 to the interphase and early mitotic centromeres. However, contrary to the simple view that the Plk1-PBIP1 complex formation is critical for mitotic functions of Plk1 (as illustrated in Fig. 1D), PBIP1 appears to function as a temporary scaffold to tightly build up and sequester Plk1 at the interphase centromeres in a manner that requires Plk1-dependent PBIP1 phosphorylation at T78. Paradoxically, Plk1 also induces PBIP1 delocalization/degradation from the early mitotic kinetochores, thus allowing a swift conveyance of Plk1 itself to other kinetochore components critical for proper M-phase progression (see Fig. 4). These findings suggest that PBIP1 functions as a conveyor belt that Plk1 can hop onto at its own self-determined pace and bring itself to other mitotic kinetochore components accumulated at the other end of the belt.

Proper recruitment and function of Plk1 at the mitotic kinetochores appear to be critical for normal chromosome congression and segregation. Failure in this process ultimately leads to the development of aneuploidy [17]. The Plk1-PBIP1 interaction exemplifies a unique mechanism involving both temporal and spatial regulation of PBIP1 at the centromeres. Proper regulation of Plk1 function at the centromeres appears to require multiple components that orchestrate timely recruitment, sequestration, and delivery of Plk1 to the right components at the mitotic kinetochores. Adding more complexity to the already complicated Plk1-PBIP1 interaction, other studies suggest that PBIP1 also forms a complex with other centromeric components such as CENP-O, CENP-P, CENP-Q, and CENP-R [21,22]. Further investigation on the regulation of PBIP1 modification, delocalization, and degradation, as well as the determination of additional components critical for PBIP1 function will be necessary to better comprehend the functions of the Plk1-PBIP1 interaction during M-phase progression.

## Competing interests

The author(s) declare that they have no competing interests.

## Authors' contributions

KSL wrote the paper. DYO, YHK, and JEP contributed the results and designed the figures. All authors read and approved the final manuscript.
